# Effects of Potassium Bicarbonate on Gel, Antioxidant and Water Distribution of Reduced-Phosphate Silver Carp Surimi Batter under Cold Storage

**DOI:** 10.3390/gels9100836

**Published:** 2023-10-21

**Authors:** Jing-Chao Fan, Guang-Hui Liu, Kai Wang, Chun Xie, Zhuang-Li Kang

**Affiliations:** 1 School of Pharmacy, Shangqiu Medical College, Shangqiu 476100, China; lgh1178@163.com (G.-H.L.); w202306292023@163.com (K.W.); xspring9090@163.com (C.X.); 2 College of Tourism and Culinary, Yangzhou University, Yangzhou 225127, China; kzlnj1988@163.com

**Keywords:** storage loss, texture property, potassium bicarbonate, total volatile basic nitrogen, total plate count

## Abstract

The changes in storage loss, water distribution status, gel characteristics, thiobarbituric acid reactive substances (TBARSs), total volatile basic nitrogen, and total plate count of cooked reduced-phosphate silver carp surimi batter during cold storage at 4 °C were investigated. The storage loss, content of free water, pH, hardness, TBARSs, total volatile basic nitrogen value, and total plate count of all cooked silver carp surimi batters significantly increased (*p* < 0.05) with an increase in cold storage time. Meanwhile, the content of immobilized water, whiteness, springiness, and cohesiveness significantly decreased (*p <* 0.05). At the same cold storage time, the sample of cooked reduced-phosphate silver carp surimi batter had lower water mobility, darker color, and better texture characteristics than the cooked silver carp surimi batter without potassium bicarbonate; however, the values of TBARSs, total volatile basic nitrogen, and total plate count were not significantly different (*p* > 0.05). This meant that there was no difference between potassium bicarbonate and sodium tripolyphosphate in antioxidant and antibacterial activity during the cold storage of silver carp surimi batter. To summarize, the use of potassium bicarbonate instead of sodium tripolyphosphate could produce cooked reduced-phosphate silver carp surimi batter with better water-holding capacity and gel characteristics during cold storage.

## 1. Introduction

Emulsified surimi products are widely accepted by consumers because they offer a wide choice of textures and flavors and high nutritional value and cost performance [1,2,3]. Most emulsified surimi products are added with phosphorus, such as sodium tripolyphosphate, sodium pyrophosphate, and sodium hexametaphosphate, to increase the solubility of salt soluble proteins, dissociate actomyosin, and then improve gel properties [4,5], which determine the quality and shelf life of the products. However, excess phosphorus can cause tooth, bone, and other diseases [6]. Thus, some consumers are increasingly paying attention to reduced-phosphate foods.

Using potassium bicarbonate to replace phosphorus in aquatic and meat products has been reported by some studies [7,8,9,10]. Xie et al. [7] found that using potassium bicarbonate to partially replace sodium tripolyphosphate can improve the processing and gel properties of silver carp batter. Li et al. [8] used potassium bicarbonate, trehalose, and chitosan to keep the property stability of tilapia fillets stored at −18 °C. They found that using a suitable concentration of potassium bicarbonate, trehalose, and chitosan can increase the tilapia fillets’ water retention capacity and quality. Jaico, Prabhakar, Adhikari, Singh, and Mohan [9] reported that adding potassium bicarbonate causes meatloaf to produce a pinkish-red color and significant tenderizing and juiciness effects, leading to an improvement in its consumption quality, texture, and sensorial attributes. Mohan et al. [10] showed that the addition of potassium bicarbonate enhances the processing and texture properties of processed ground beef. Lee et al. [11] reported that potassium bicarbonate is a healthier phosphorus substitute, which improves the quality of marinated chicken breast meat. Our previous studies reported that using potassium bicarbonate to partially/totally replace sodium tripolyphosphate causes the cooking yield, hardiness, springiness, chewiness, storage modulus, and β-sheet and β-turn structure content of silver carp batter to significantly increase, which also improves the gel and rheology characteristics [7]. However, the difference between the effect of potassium bicarbonate and sodium tripolyphosphate on the quality changes in cooked silver carp surimi during cold storage is not clear; the water-holding capacity, gel properties, microbes, and thiobarbituric acid reactive substances (TBARSs) especially need further research. Therefore, based on the above, this study’s aim was to analyze the effect of potassium bicarbonate on the water stability, whiteness, texture properties, TBARSs, total volatile basic nitrogen, and total plate count of cooked reduced-phosphate silver carp surimi batter stored at 4 °C for 7 days.

## 2. Results and Discussion

### 2.1. Storage Loss

**Figure 1:**
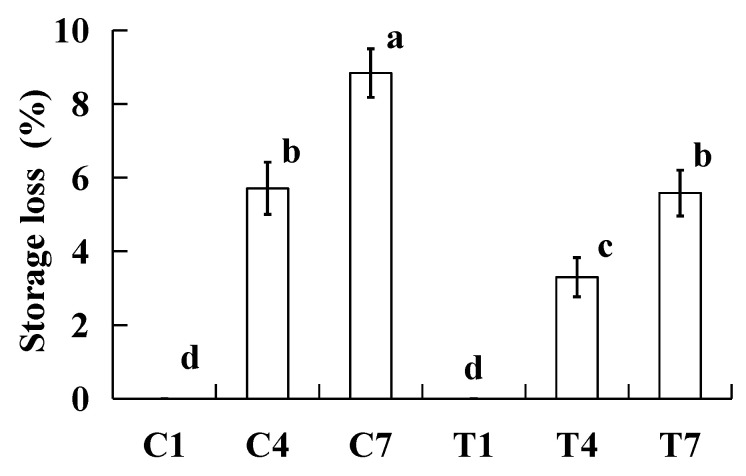
Storage loss effect of the cooked silver carp surimi batters (%) observed with various amounts of potassium bicarbonate and sodium tripolyphosphate during cold storage. C1, 0.4 g potassium bicarbonate and stored on the 1st day; C4, 0.4 g potassium bicarbonate and stored on the 4th day; C7, 0.4 g potassium bicarbonate and stored on the 7th day; T1, 0.3 g potassium bicarbonate, 0.1 g sodium tripolyphosphate, and stored on the 1st day; T4, 0.3 g potassium bicarbonate, 0.1 g sodium tripolyphosphate, and stored on the 4th day; T7, 0.3 g potassium bicarbonate, 0.1 g sodium tripolyphosphate, and stored on the 7th day. Each value represents the mean ± SD, *n* = 4. ^a–d^ Different parameter superscripts in the figure indicate significant differences (*p <* 0.05).

Storage loss reflects the stability of water and fat in cooked surimi products during storage and affects the product quality. The effect of the storage loss of cooked silver carp surimi batter was made with various amounts of potassium bicarbonate and sodium tripolyphosphate during cold storage, as presented in Figure 1. All the storage losses of cooked silver carp surimi batter significantly increased (*p <* 0.05) with an increase in the storage time. The reason for this is that the protein and fat of cooked silver carp surimi batter were oxidized, leading to the gel network structure being destroyed during the cold storage and a decrease in the water retention capacity [12]. In addition, the growth and propagation of microorganisms during cold storage can damage the gel structure and increase the loss of water and oil [13]. At the same storage time, the storage loss of cooked silver carp surimi batter without potassium bicarbonate significantly increased (*p <* 0.05) compared to that of the sample with potassium bicarbonate. The samples of C4 and T7 had the same storage loss. Some researchers reported that potassium bicarbonate and sodium tripolyphosphate increase the pH of a water solution, whereas the buffering capacity of potassium bicarbonate is better than that of sodium tripolyphosphate [14]. As a result, the sample with potassium bicarbonate had a better water- and fat-holding capacity and lower storage loss [10]. Our previous study found that the processing characteristics of silver carp surimi batter are improved when potassium bicarbonate is used to replace sodium tripolyphosphate [7]. Hence, the addition of potassium bicarbonate increased the stability of water in the cooked silver carp surimi batter during cold storage. 

### 2.2. Low-Field NMR

Low-field NMR is a rapid and nondestructive method of determining moisture and fluidity in muscle and aquatic products [15,16]. The changes in T2 relaxation of the cooked silver carp surimi batters were made with various amounts of potassium bicarbonate and sodium tripolyphosphate during cold storage as shown in Table 1. All samples showed three peaks, named T_2b_ (bond water, 0–10 ms), T_21_ (immobilized water, 10–100 ms), and T_22_ (free water, 100–1000 ms), respectively [17]. Therein, T_2b_ represents the water tightly associated with protein and macro-molecular constituents [18]. T_21_ represents the intra-myofibrillar water and water within the protein structure [19]. T_22_ represents the extra-myofibrillar water, which flows easily during processing and storage [20]. Compared with the samples without potassium bicarbonate, the initial relaxation times of T_2b_, T_21_, and T_22_ in the samples with potassium bicarbonate were shorter (*p <* 0.05) at the same cold storage time, and that meant that the water in the samples with potassium bicarbonate had a closer connection. As is known to all, the T_2_ reflects the fluidity of water laterally, a longer T_2_ means a higher fluidity [19]. Meanwhile, the peak ratios of P_2b_ and P_22_ in the samples with potassium bicarbonate declined (*p <* 0.05), and the P_21_ was increased (*p <* 0.05) at the same cold storage time compared with the samples without potassium bicarbonate. On the other hand, with increasing the cold storage time, the initial relaxation times of T_21_ and T_22_ in all samples significantly increased (*p <* 0.05), accompanied by the peak ratio of P_22_ being significantly increased (*p <* 0.05) and the P_21_ significantly decreased (*p <* 0.05). These results meant that adding potassium bicarbonate lowered the fluidity of water in the cooked silver carp surimi batters during cold storage.

### 2.3. pH

**Figure 2:**
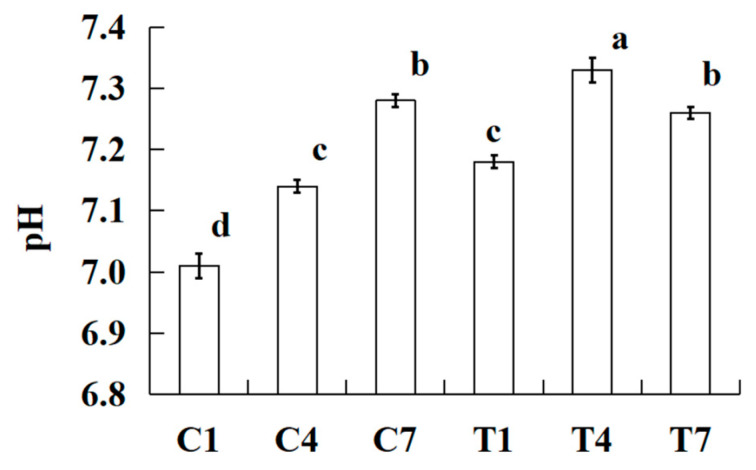
Effect on the cooked silver carp surimi batters regarding pH observed with various amounts of potassium bicarbonate and sodium tripolyphosphate during cold storage. C1, 0.4 g potassium bicarbonate and stored on the 1st day; C4, 0.4 g potassium bicarbonate and stored on the 4th day; C7, 0.4 g potassium bicarbonate and stored on the 7th day; T1, 0.3 g potassium bicarbonate, 0.1 g sodium tripolyphosphate, and stored on the 1st day; T4, 0.3 g potassium bicarbonate, 0.1 g sodium tripolyphosphate, and stored on the 4th day; T7, 0.3 g potassium bicarbonate, 0.1 g sodium tripolyphosphate, and stored on the 7th day. Each value represents the mean ± SD, *n* = 4. ^a–d^ Different parameter superscripts in the figure indicate significant differences (*p <* 0.05).

The changes in pH of the cooked silver carp surimi batters were made with various amounts of potassium bicarbonate and sodium tripolyphosphate during cold storage as shown in Figure 2. The pH of all cooked silver carp surimi batters significantly increased (*p <* 0.05) with the increase in cold storage time, except for the sample of T7. It is possible that microbial growth and reproduction utilize the proteins in cooked silver carp surimi batters to generate alkaline nitrogen-containing compounds during cold storage, such as amines and trimethylamine, resulting in an increase in pH [21,22,23]. In addition, previous studies found that lipid oxidation and protein oxidation produce alkaline substances, which improve the pH of batters during cold storage [24,25]. Especially, because the samples with potassium bicarbonate had a higher pH than samples without potassium bicarbonate, after 4 days of cold storage, there was a certain degree of decreasing in pH caused by bacterial metabolism utilizing organic small molecules for fermentation and acid production [26].

### 2.4. Whiteness

**Figure 3:**
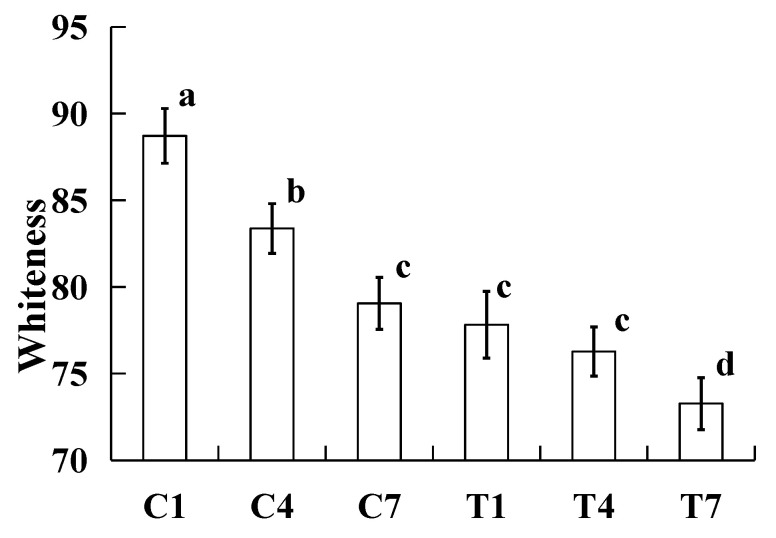
Effect on the cooked silver carp surimi batters regarding whiteness observed with various amounts of potassium bicarbonate and sodium tripolyphosphate during cold storage. C1, 0.4 g potassium bicarbonate and stored on the 1st day; C4, 0.4 g potassium bicarbonate and stored on the 4th day; C7, 0.4 g potassium bicarbonate and stored on the 7th day; T1, 0.3 g potassium bicarbonate, 0.1 g sodium tripolyphosphate, and stored on the 1st day; T4, 0.3 g potassium bicarbonate, 0.1 g sodium tripolyphosphate, and stored on the 4th day; T7, 0.3 g potassium bicarbonate, 0.1 g sodium tripolyphosphate, and stored on the 7th day. Each value represents the mean ± SD, *n* = 4. ^a–d^ Different parameter superscripts in the figure indicate significant differences (*p <* 0.05).

The effects on the whiteness of the samples were made with various amounts of potassium bicarbonate and sodium tripolyphosphate during cold storage as shown in Figure 3. All whiteness values of cooked silver carp surimi batters significantly increased (*p <* 0.05) with the increase in storage time, except the samples of T1 and T4. The changes in whiteness were caused by light, and more water on the surface of cooked samples caused the increase in lightness and whiteness [27]. In this study, water was lost during cold storage (Figure 1), thus, the whiteness was lowered with the increase in storage time. In addition, due to the samples with potassium bicarbonate having a darker color and increasing pH (Figure 2) lowering the oxidation rate of myoglobin to metmyoglobin [7], the changes in whiteness between the samples of the 1st and 4th days were not significantly different (*p* > 0.05). This result indicated that the color of the samples with potassium bicarbonate was more stable than that of the samples without potassium bicarbonate. 

### 2.5. Texture Properties

The effects on texture properties of the samples were made with various amounts of potassium bicarbonate and sodium tripolyphosphate during cold storage as shown in Table 2. All hardness values of cooked samples significantly increased (*p <* 0.05) with the increase in cold storage time, which is because the moisture content of cooked silver carp surimi batters was decreased (Figure 1), which led to the hardness value increasing [28]. Meanwhile, the springiness values between the samples of the 1st and 4th days were not significantly different (*p* > 0.05), and then significantly decreased (*p <* 0.05) in the sample of the 7th day; the cohesiveness values significantly decreased (*p <* 0.05) with the increase in cold storage time. The result was caused by the increase in fat oxidation, microbial activity, pH, and water loss [29]. In addition, the chewiness values of samples with/without potassium bicarbonate were not significantly different (*p* > 0.05) with the increase in cold storage time, the main reason for which is that the hardness values increased significantly with the increase in cold storage time. At the same cold storage time, the texture properties of the samples with potassium bicarbonate were better than those of samples without potassium bicarbonate, indicating that the texture properties of the samples with potassium bicarbonate were more stable.

### 2.6. TBARS

**Figure 4:**
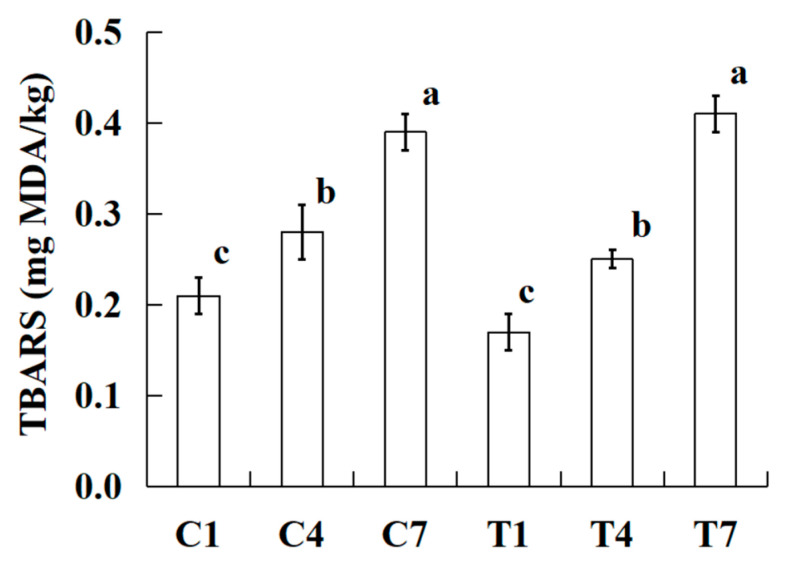
Effect on the cooked silver carp surimi batters regarding TBARSs (mg MDA/kg) observed with various amounts of potassium bicarbonate and sodium tripolyphosphate during cold storage. C1, 0.4 g potassium bicarbonate and stored on the 1st day; C4, 0.4 g potassium bicarbonate and stored on the 4th day; C7, 0.4 g potassium bicarbonate and stored on the 7th day; T1, 0.3 g potassium bicarbonate, 0.1 g sodium tripolyphosphate, and stored on the 1st day; T4, 0.3 g potassium bicarbonate, 0.1 g sodium tripolyphosphate, and stored on the 4th day; T7, 0.3 g potassium bicarbonate, 0.1 g sodium tripolyphosphate, and stored on the 7th day. Each value represents the mean ± SD, *n* = 4. ^a–c^ Different parameter superscripts in the figure indicate significant differences (*p <* 0.05).

The TBARS values of the samples were induced with various amounts of potassium bicarbonate and sodium tripolyphosphate during cold storage as shown in Figure 4. At the same cold storage time, all TBARS values of cooked silver carp surimi batters were not significantly different (*p* > 0.05). Meanwhile, all TBARS values significantly increased (*p <* 0.05) with the increase in cold storage time. It is well known that the TBARS value is a widely used indicator for lipid oxidation in aquatic products and processed aquatic products. There is a strong correlation between TBARS value and the degree of fat oxidation in products. The higher the TBARS value, the higher the degree of fat oxidation, while the more severe the rancidity and the more small molecule substances (aldehydes, ketones, acids, etc.) produced [30,31]. The result indicated that more fat oxidation was produced during cold storage and more water was washed out to form free water (Figure 1 and Table 1). The free water favored the formation of free radicals in the cooked silver carp surimi batter and then increased the TBARS value [32]. 

### 2.7. Total Volatile Basic Nitrogen

Total volatile basic nitrogen indicates the spoilage and deterioration of aquatic products and it reflects the accumulation of volatile ammonia in aquatic product proteins due to decomposing proteins and nonprotein substances [33]. The lower its content, the higher the freshness of aquatic products. The total volatile basic nitrogen values of the samples were induced with various amounts of potassium bicarbonate and sodium tripolyphosphate during cold storage as shown in Table 3. All the total volatile basic nitrogen values of cooked silver carp surimi batters were not significantly different (*p* > 0.05) at the same cold storage time. Meanwhile, all values increased significantly (*p <* 0.05) with the increase in cold storage time. It is possible that microorganisms grow rapidly at this stage, causing the total volatile basic nitrogen value to increase rapidly [34]. Guan et al. [35] found that the total volatile basic nitrogen of hairtail fish balls significantly increased when the balls were stored at 4 °C from 1 to 15 days. Thus, the use of potassium bicarbonate instead of sodium tripolyphosphate has no significant effect on the total volatile basic nitrogen value of cooked silver carp surimi batter.

### 2.8. Total Plate Count

The total plate counts of the samples were measured with various amounts of potassium bicarbonate and sodium tripolyphosphate during cold storage as shown in Table 3. The total plate counts of cooked silver carp surimi batters were not significantly different (*p* > 0.05) at the same cold storage time. Meanwhile, all the total plate counts significantly increased (*p <* 0.05) with the increase in cold storage time. The reason is possibly that all samples had a similar storage loss (Figure 1) and pH (Figure 2), the higher apparent moisture of samples increased the A_w_, and the result promoted the growth and reproduction of microorganisms [36,37]. Solo-de-Zaldívar et al, [38] reported that the total viable bacterial counts of restructured fish muscle products were gradually increased during chilled storage from 0 to 35 days. The result showed that high pH and total volatile basic nitrogen values were beneficial for the growth of microorganisms during cold storage. 

## 3. Conclusions

At the same cold storage time, the use of potassium bicarbonate instead of sodium tripolyphosphate has a significant effect on the storage loss, initial relaxation time, peak ratio, pH, whiteness, hardness, springiness, and cohesiveness of cooked silver carp surimi batters but has no significant effect on the chewiness, TBARSs, total volatile basic nitrogen value, and total plate count of cooked silver carp surimi batters. The storage loss, T_2b_, T_21_, T_22_, and P_22_ of the samples with potassium bicarbonate had lower values than those of the samples without potassium bicarbonate and they had higher values of pH and texture properties. With the increase in cold storage time, the storage loss, T_2b_, T_21_, T_22_, P_22_, pH, hardness, TBARSa, total volatile basic nitrogen value, and total plate count significantly increased, and the whiteness, springiness, and cohesiveness values decreased significantly. From the above, the cold storage performances of cooked silver carp surimi batter with potassium bicarbonate could be improved.

## 4. Materials and Methods

### 4.1. Materials and Silver Carp Surimi Batters Prepared

According to the method of our previous study [7], fresh silver carp and pork back-fat were purchased from a farmer’s market in Shangqiu City (China). Referring to the method of An et al. [39], the silver carp surimi was prepared. Potassium bicarbonate, sodium tripolyphosphate, and sodium chloride (analytically pure) were purchased from Tianjin Boddi Chemical Co. Ltd., (Tianjin, China). White pepper, garlic powder, and sugar were purchased from a local market (Shangqiu, China). All other reagents were analytically pure.

The formulas of raw silver carp surimi batters were as follows: silver carp surimi 100 g, pork back-fat 20 g, ice water 20 g, white pepper 1 g, garlic powder 1.5 g, sugar 3.5 g, sodium chloride 1.5 g. C contained sodium tripolyphosphate 0.4 g; T contained potassium bicarbonate 0.3 g and sodium tripolyphosphate 0.1 g. According to our previous study [7], the batter was produced, vacuum packed, and stored at 4 °C.

### 4.2. Cold Storage

All cooked cooked silver carp surimi batters were stored at 4 °C for 7 days. The samples of C stored for 1, 4, and 7 days were named C1, C4, and C7; the samples of T stored for 1, 4, and 7 days were named T1, T4, and T7, respectively.

### 4.3. Determination of Storage Loss

Storage loss of cooked silver carp surimi batter was determined on the 1st, 4th, and 7th days, respectively. The sample with casing was weighed (original sample). Then, the water was removed and the sample was reweighed (reweighed sample). The calculation formula of storage loss wa showed as follows:

Storage loss (%) = (original sample − reweighed sample)/original sample × 100

### 4.4. Low-Field NMR Measurement

According to the method of our previous study [20], the sample was cut into about 2 g cubes and placed in Ziploc bags, and the low-field NMR relaxation measurement was performed by a Niumag NMR analyzer (NMI20-040 V-1, Shanghai, China). During the process of measurement, a resonance frequency of 22.6 MHz at 32 °C was operated, the value of τ (the time between 90° and 180° pulse) was 200 μs, the number of collected echoes was 5000, and the scan was repeated 32 times with a repetition interval of 6.5 s to obtain 12,000 echoes. 

### 4.5. pH Measurement

Twenty grams of cooked silver carp surimi batter and 80 mL distilled water were homogenized at 15,000 rpm for 10 s on the 1st, 4th, and 7th days, respectively. Then, pH was determined using a pH meter.

### 4.6. Whiteness Measurement

The color of the cooked silver carp surimi batter core was determined by a colorimeter (Minolta, Japan) on the 1st, 4th, and 7th days. The calculation formula of whiteness was as follows:

Whiteness = 100 − [(100 − L^*^)^2^ + a^*2^+ b^*2^]^1/2^

### 4.7. Texture Properties Measurement

According to the method of our previous study [7], the texture properties of cooked silver carp surimi batter were measured and analyzed. Briefly, the sample was left at 20 °C for 2 h, then a cylinder (diameter, 15 mm; height, 20 mm) was cut and the texture profile analysis was carried out by a P/36R probe (Stable Micro System Ltd., Surrey, UK) with the post-test speed of 2.0 mm/s and strain of 50%.

### 4.8. TBARS Measurement

The TBARSs of cooked silver carp surimi batter were determined by referring to the method of Ulu [40]. Briefly, a 10 g sample was crushed, then 50 mL 7.5% (*w*/*v*) trichloroacetic acid was added. After shaking for 30 min, the sample solution was filtered with three layers of filter paper. A 5 mL volume of filtrate was added to 5 mL 2-thiobarbituric acid (0.02 M) and left in a boiling water bath for 40 min. After the reaction solution was cooled to room temperature (25 °C), it was centrifuged at 5,500 r/min for 25 min. At 532 nm, the supernatant’s absorbance was gauged. The results were calculated from the standard curve prepared with 1,1,3,3-tetramethoxypropane and expressed by the amount of malondialdehyde per kilogram (mg MDA/kg).

### 4.9. Total Volatile Basic Nitrogen Measurement

According to the method of AOAC (2011), the total volatile basic nitrogen of samples was measured.

### 4.10. Total Plate Count Measurement

According to the method of AOAC (2011), the total plate count of samples was measured.

### 4.11. Statistical Analysis

In each replication, the batters of different storage times were prepared and 160 cooked silver carp surimi batters (different potassium bicarbonate and sodium tripolyphosphate contents) were used. The data were analyzed by a one-way ANOVA and GLM procedure (SPSS v.20.0, SPSS Inc., Chicago, USA). Differences between means were identified using the LSD procedure and considered significant at *p <* 0.05.

## Figures and Tables

**Table 1:** Effect on the cooked silver carp surimi batters regarding initial relaxation time (ms) and peak ratio (%) observed with various amounts of potassium bicarbonate and sodium tripolyphosphate during cold storage.

Sample	Initial Relaxation Time (ms)			Peak Ratio (%)		
	T_2b_	T_21_	T_22_	P_2b_	P_21_	P_22_
C1	2.32 ± 0.10 ^a^	46.56 ± 1.26 ^c^	465.42 ± 12.56 ^c^	1.38 ± 0.17 ^a^	90.26 ± 0.87 ^b^	8.58 ± 0.46 ^d^
C4	2.41 ± 0.09 ^a^	57.48 ± 1.18 ^b^	498.23 ± 11.27 ^b^	1.42 ± 0.22 ^a^	86.75 ± 0.76 ^c^	11.15 ± 0.39 ^c^
C7	2.47 ± 0.12 ^a^	68.15 ± 1.30 ^a^	536.70 ± 12.04 ^a^	1.50 ± 0.26 ^a^	81.58 ± 0.93 ^d^	17.49 ± 0.33 ^a^
T1	1.92 ± 0.12 ^b^	35.51 ± 1.27 ^d^	336.27 ± 12.33 ^e^	0.96 ± 0.15 ^b^	93.32 ± 0.91 ^a^	6.37 ± 0.35 ^e^
T4	2.07 ± 0.11 ^b^	47.22 ± 1.31 ^c^	387.81 ± 11.48 ^d^	1.04 ± 0.20 ^b^	90.47 ± 0.83 ^b^	8.50 ± 0.39 ^d^
T7	2.11 ± 0.13 ^b^	55.36 ± 1.23 ^b^	448.87 ± 12.19 ^c^	0.99 ± 0.19 ^b^	85.44 ± 0.95 ^c^	13.67 ± 0.44 ^b^

C1, 0.4 g potassium bicarbonate and stored on the 1st day; C4, 0.4 g potassium bicarbonate and stored on the 4th day; C7, 0.4 g potassium bicarbonate and stored on the 7th day; T1, 0.3 g potassium bicarbonate, 0.1 g sodium tripolyphosphate, and stored on the 1st day; T4, 0.3 g potassium bicarbonate, 0.1 g sodium tripolyphosphate, and stored on the 4th day; T7, 0.3 g potassium bicarbonate, 0.1 g sodium tripolyphosphate, and stored on the 7th day. Each value represents the mean ± SD, *n* = 4. ^a–e^ Different parameter superscripts in the figure indicate significant differences (*p <* 0.05).

**Table 2:** Effect on the cooked silver carp surimi batters regarding texture properties observed with various amounts of potassium bicarbonate and sodium tripolyphosphate during cold storage.

Sample	Hardness (N)	Springiness	Cohesiveness	Chewiness (N·mm)
C1	47.37 ± 1.27 ^d^	0.853 ± 0.011 ^c^	0.613 ± 0.011 ^c^	24.71 ± 1.27 ^b^
C4	51.33 ± 1.35 ^c^	0.827 ± 0.009 ^c^	0.595 ± 0.007 ^d^	25.24 ± 1.04 ^b^
C7	57.25 ± 1.16 ^b^	0.771 ± 0.012 ^d^	0.557 ± 0.009 ^e^	24.58 ± 1.16 ^b^
T1	53.91 ± 1.13 ^c^	0.911 ± 0.012 ^a^	0.670 ± 0.009 ^a^	31.10 ± 1.21 ^a^
T4	56.02 ± 1.27 ^b^	0.880 ± 0.015 ^a^	0.642 ± 0.010 ^b^	31.63 ± 1.37 ^a^
T7	59.87 ± 1.21 ^a^	0.844 ± 0.010 ^b^	0.615 ± 0.012 ^c^	30.92 ± 1.16 ^a^

C1, 0.4 g potassium bicarbonate and stored on the 1st day; C4, 0.4 g potassium bicarbonate and stored on the 4th day; C7, 0.4 g potassium bicarbonate and stored on the 7th day; T1, 0.3 g potassium bicarbonate, 0.1 g sodium tripolyphosphate, and stored on the 1st day; T4, 0.3 g potassium bicarbonate, 0.1 g sodium tripolyphosphate, and stored on the 4th day; T7, 0.3 g potassium bicarbonate, 0.1 g sodium tripolyphosphate, and stored on the 7th day. Each value represents the mean ± SD, *n* = 4. ^a–d^ Different parameter superscripts in the figure indicate significant differences (*p <* 0.05).

**Table 3:** Effect on the cooked silver carp surimi batters regarding total volatile basic nitrogen (mg/100 g) and total plate count (CFU/g) observed with various amounts of potassium bicarbonate and sodium tripolyphosphate during cold storage.

Sample	Total Volatile Basic Nitrogen (mg/100 g)	Total Plate Count (CFU/g)
C1	3.34 ± 0.32 ^c^	3.23 × 10
C4	8.50 ± 0.39 ^b^	6.26 × 10^2^
C7	17.31 ± 0.52 ^a^	5.31 × 10^4^
T1	3.63 ± 0.44 ^c^	4.60 × 10
T4	9.30 ± 0.38 ^b^	5.52 × 10^2^
T7	16.68 ± 0.71 ^a^	4.63 × 10^4^

C1, 0.4 g potassium bicarbonate and stored on the 1st day; C4, 0.4 g potassium bicarbonate and stored on the 4th day; C7, 0.4 g potassium bicarbonate and stored on the 7th day; T1, 0.3 g potassium bicarbonate, 0.1 g sodium tripolyphosphate, and stored on the 1st day; T4, 0.3 g potassium bicarbonate, 0.1 g sodium tripolyphosphate, and stored on the 4th day; T7, 0.3 g potassium bicarbonate, 0.1 g sodium tripolyphosphate, and stored on the 7th day. Each value represents the mean ± SD, *n* = 4. ^a–c^ Different parameter superscripts in the figure indicate significant differences (*p <* 0.05).

## Data Availability

Not applicable.
